# Buccinator myo-mucosal and mucoperiosteal flap opposite rotation to repair anterior palatal fistula in children

**DOI:** 10.1186/s12903-026-08947-7

**Published:** 2026-06-18

**Authors:** Weimin Shen, Jie Cui, Jianbin Chen, Jun Yan

**Affiliations:** Department of Plastic Surgery, Children’s Hospital of Nanjing Medical University, Nanjing, 210008 China

**Keywords:** Palatal fistula, Buccinator myo-mucosal flap, Mucoperiosteal flap, Palatal cleft, Pediatric

## Abstract

**Objective:**

This study sought to investigate the impact of opposite rotation of the buccinator myo-mucosal flap (BMMF) and Mucoperiosteal flap (MPF) in the repair of anterior palatal fistula (APF).

**Methods:**

A retrospective analysis was conducted on 28 pediatric patients who underwent palatal fistula repair surgery at Nanjing Children’s Hospital from December 2011 to January 2025. The patients were divided into two groups. One group of 18 cases used the opposite rotation of the BMMF and MPF to repair the APF, while the other group of 10 cases used the double flap method to repair the APF. The design of opposite rotation of the BMMF and MPF involved placing the BMMF buccal to the side of the fistula, while the MPF near the fistula was elevated and moved to the mucosa beside the fistula to fill the defect left due to the transfer of the BMMF. Simultaneously, the BMMF was transposed to the palate to repair the APF.

**Results:**

In all 28 cases, the wounds exhibited successful healing, and the colour of the distal ends of BMMF and MPF appeared normal. The follow-up period was 8 to 12 months. There were 3 cases of bleeding, 5 cases of infection, 4 cases of recurrence, and 3 cases of incision dehiscence. The palatal shape of the children showed satisfactory recovery, and postoperative pronunciation displayed varying degrees of improvement.

**Conclusion:**

Opposite rotation of BMMF and MPF represents an novel method for repairing APF following cleft palate surgery, demonstrating beneficial clinical outcomes.

## Introduction

Successful cleft palate surgery aims to restore normal anatomical morphology and physiological function to the palate. However, postoperative palatal fistulas caused by various clinical factors is one of the common complications in cleft palate surgeries.

Common fistulas are situated in the hard palate (60%) or at the junction of the soft and hard palate (33%) [1, 2]. Repairing fistulas in the anterior part of the palate presents significant challenges for plastic surgeons. Secondary repair is particularly arduous due to limited mucoperiosteal flap mobility, scarring, inadequate blood supply, and tissue volume, among other factors. Presently, commonly utilised repair methods involve local flaps around the fistulas, such as Honnebier’s local mucoperiosteal flap [3] and Rauso’s Pedicled palatal flap [4], as well as the pedicled Buccinator Myo-Mucosal Flap in the anterior region. Each method possesses its advantages and disadvantages. The more times a palatal fistulas is repaired, the more difficult the repair becomes, as numerous scars will form locally. The first issue is the difficulty in separation, and the second is the difficulty in the healing of the scar tissue. How to prevent these problems from occurring? The solution is to use normal tissues from other parts to transfer as skin flaps for repairing the fistulas.

In this study, we employed opposite rotation of the BMMF and MPF to repair fistula in the anterior palate, achieving satisfactory therapeutic results. The details are reported as follows.

## Materials and methods

### Study design and patients

This is a retrospective study that retrospectively analyzed the treatment outcomes of 28 children (16 males and 12 females) with APF who were admitted to the Children’s Hospital of Nanjing Medical University. Anterior palate fistula was identified, and parental consent for utilization was obtained. We divided it into two groups. One group used the rotation of the BMMF and MPF; the other group was the control group, using the double-flap technique methods. The inclusion criteria were to combine the two methods and inform the parents, allowing them to make a voluntary choice of the surgical method. This is a retrospective study, and since the selection method for the groups was not a randomized control group, there is a certain degree of selection bias. The clinical manifestations of the two groups are shown in Table 1. Inclusion criteria of the research group: (1) type of fistula༚V, VI, and VII of Pittsburgh fistula classification [5]. (2) fistula size: 1.0 × 1.0-2.5 × 2cm2. (3) number of previous surgeries༚2–4. (4) aged between 3 and 13 years. Inclusion criteria of the control group, (1) type of fistula༚V, VI, and VII, (2) fistula size༚1.0 × 0.8-2.2 × 2 cm2. (3) number of previous surgeries༚2–3. ༈4༉ aged between 3 and 10 years, Exclusion criteria: (1) fistula in other areas of the upper palate. (2) Patients who have undergone more than 4 surgeries. (3) There was anemia and malnutrition. The study adhered to the principles of the Declaration of Helsinki concerning research involving human subjects and received approval from the Ethics Committee of the Children’s Hospital of Nanjing Medical University ( NO༚2021-IRB-304).

**Table 1:** Patient demographic data of two groups

Surgery method	research group	control group	Total	*p*-value
No. of patients	18	10		
Sex				0.82
Male	10	6	16	
Female	8	4	12	
severity of cleft palate Before the first surgery				
Bilateral Ⅲ	18	10	28	
This time, the age at which the fistula was repaired				0.90
Average	8.2	8.9	8.55	
Range	6–12	8–13	6–13	
Age at time of the first surgery(month)				0.00122
Average	10.5	10.2	20.7	
Range	10–12	8–13	8–13	
fistula size,				0.33
Average	2.5	2	2.25	
Range	1.0 × 1.0-2.5 × 2	1.0 × 0.8-2.2 × 2	0.8–4.5	
number of previous surgeries				0.42
Average	3.5	3	6.5	
Range	2–4	2–3	2–4	
Type of fistula (Pittsburgh classification systems)				0.13
type V	6	3	9	
type VI	4	3	7	
type VII	8	4	12	
Follow-up duration(month)				0.22
Average	9	12	21	
Range	6–12	10–14	6–14	

*P* < 0.01, There was a statistically significant difference in the age at the first surgical treatment

The remaining items: *P* > 0.01, No significant difference

### Operative technique

**Fig. 1:**
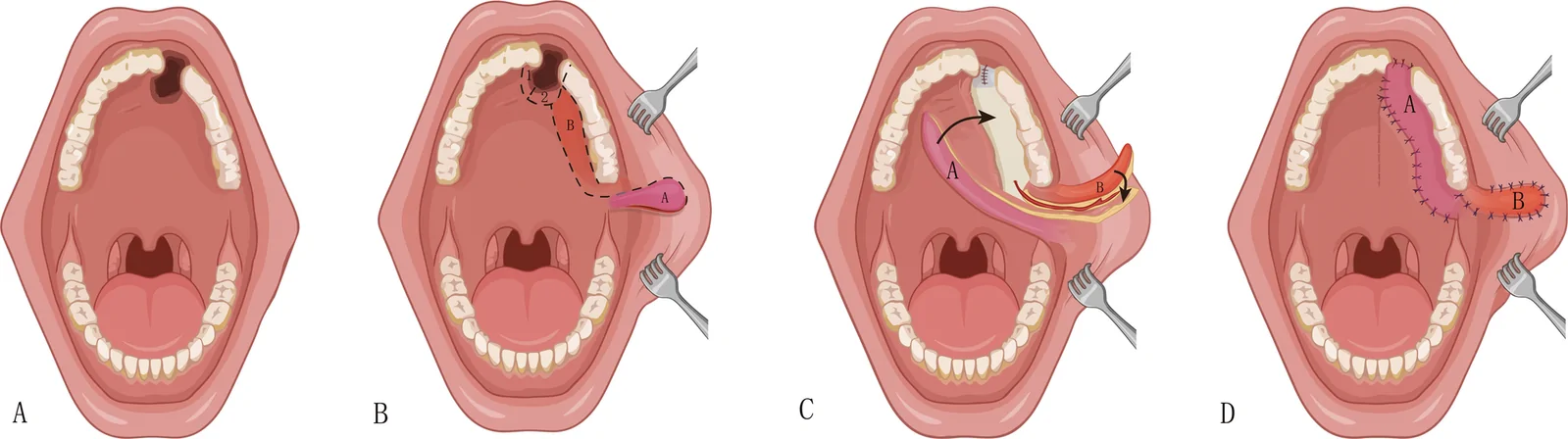
The surgical design is shown. **A** Design of buccal mucosa and Mucoperiosteal flap opposite rotation: (**A**) Diagram of anterior palatal fistula. **B** pedicled buccinator myo-mucosal flap A was designed on the buccal mucosa of the fistula side, allowing its length to be transferred to the anterior palatal fistula. The aspect ratio of the flap is 1:3. The flap originate buccal to and centered on the retromolar trigone between the maxillary and mandibular dentition, extending up toward the commissure as needed and preferentially avoiding Stensen’s duct. The flap B was Mucoperiosteal flap and is an island-shaped flap with a large palatine artery. **C** Intraoperative flap rotation. **D** Flap opposite after rotation

**Fig. 2:**
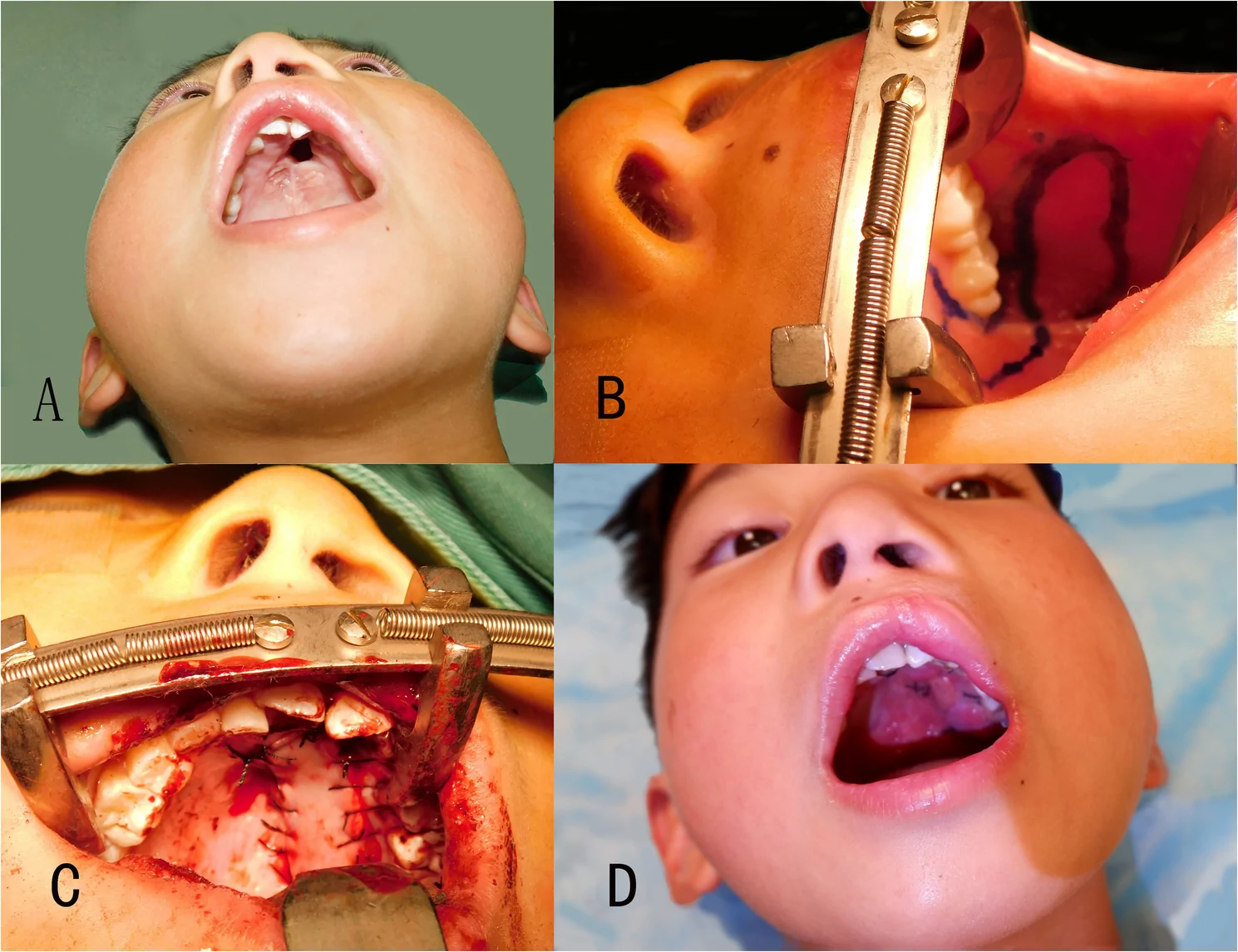
Surgical design. **A** Presentation of Anterior Palatal Fistula. **B** Design of Buccal Mucosa and Mucoperiosteal flap Opposite Rotation. The tip of the buccal myo-mucosal flap is customized to the defect stencil template, and the arc of rotation is calculated from the buccal edge of the retromolar trigone to facilitate tension-free inset. **C** Conclusion of Surgical Repair **D** Satisfactory Healing of the Repair Site One Month Postoperatively

The surgical method of the BMMF and MPF opposite rotation technique is as follows: ①. The surgical design is shown in Fig. 1A, B, C and D. ②. Mucoperiosteal flap 1 was fashioned on one side of the fistula hole, with its pedicle connected to the nasal mucosa at the fistula ‘s edge. ③. Mucoperiosteal flap 2 was created on the opposite side of the fistula, with its pedicle connected to the nasal mucosa behind the contralateral margin of the fistula. ④. The two flaps were turned into the nasal cavity and the nasal fistula was closed with sutures. ⑤. A pedicled buccinator myo-mucosal flap A was designed on the buccal mucosa of the fistula side, allowing its length to be transferred to the anterior palatal fistula. The aspect ratio of the flap is 1:3. If the distance to the fistula point is 45 mm, then the width of the pedicle of the buccal muscle mucosal flap will be 15 mm. The flap originate buccal to and centered on the retromolar trigone between the maxillary and mandibular dentition, extending up toward the commissure as needed and preferentially avoiding Stensen’s duct. The flap A included buccinator muscle. It is a localized muscular-mucosal flap. The tip is customized to the stencil template, and the arc of rotation is calculated from the buccal edge of the retromolar trigone to facilitate tension-free inset. ⑥. A mucoperiosteal composite flap B was created. This flap B is an island-shaped flap with a large palatine artery. We dissected the flap B from the upper alveolar mucosa, extending from the fistula to the molars, carefully separating the palatine major artery, Isolated about 1 cm, and lifting the flap to transfer it to the buccal region to repair the majority of the defect left after the removal of the buccal muscle mucosal flap. Simultaneously, the BMMF was transferred to repair the anterior palatal fistula. ⑦. The three flaps were rotated inward, covering the first two flaps, and secured together with longitudinal mattress sutures to close the fistula hole. Please refer to Fig. 2A, B, C and D for illustration.

### Postoperative management

Following surgery, antibiotics were administered routinely to prevent infection and commonly used Cefoxitin Sodium (an anaerobic bacterial antibiotic), 1 g per dose, once every 6 to 8 h. The oral cavity was cleansed twice daily by healthcare personnel. A liquid diet was adhered to for 3 weeks, followed by a semi-liquid diet for an additional week. After 1 month, patients resumed a general diet.

### Statistical analysis

We used the SPSS software for statistical analysis. All our cases were treated by the same doctor. The study divided the participants into two groups. One group was the opposite rotation of the BMM and MPF, and the other group used the double flap method to repair the APF. Categorical data were assessed with Fisher’s exact test. The normality of distribution was assessed using the Shapiro–Wilk test in this study, and continuous variables were compared by using independent two-sample t-tests or Mann–Whitney U tests, as appropriate. Statistical analysis indicates that there was a significant difference between the two groups in terms of recurrent palatal fistula rate and incision rupture. There were no significant differences in infection, bleeding and pronunciation improvement compared with other methods, refer to Tables 1 and 2.

**Table 2:** Statistical analysis of complications from opposite rotation of BMMF + MPF and double-flap methods

	opposite rotation of BMMF and MPF	double-flap methods	Total	*p*-value
complications				0.59
infection	1	4	5	
bleeding	2	1	3	
Recurrence				0.33
Recurrence	1	3	4	
incision rupture	1	2	3	

*P* > 0.05, No significant difference

## Results

This study involved 28 cases of anterior palatal clefts. The patients ranged in age from 3 to 13 years old, with an average age of 8.55 years. In the research cohort, there were 8 cases of anterior left palatal fistula, 7 cases of anterior right palatal fistula, and 3 cases of anterior midline palatal fistula. According to the Pittsburgh palatal fistula classification method, there were 6 cases of type V, 4 cases of type VI, and 8 cases of type VII. The size range of the perforations was 1.0 × 1.0 cm² to 2.5 × 2 cm², all of which were large fistulas. All 18 cases in research group presented with bilateral complete cleft palate, with 11 cases repaired by Langenbeck’s method and 7 by Furlow’s Z plasty. Prior to fistula repair, 4 cases had undergone two fistula repair, and 14 cases had undergone two fistula repairs. The number of preoperative repair procedures was 2 to 5 times, with an average of 3.5 times. The age at which these children underwent their first palate cleft surgery was within 12 months. In all the cases of the research group, the greater palatine artery was not ruptured or severed during the separation process. After the operation, the blood supply of the bone-mucosa island flap was good. Among the 10 patients in the control group, there were 4 cases of anterior left palatal fistula, 3 cases on the right side, and 3 cases at the midline. There were 3 cases of type V, 3 cases of type VI, and 4 cases of type VII. The size of the perforation ranged from 1.0 × 0.8 cm² to 2.2 × 2 cm². All were large fistulas. The control group used the double flap method to correct the palatal fistula. 10 cases were after bilateral cleft palate surgery and the age of the first cleft palate repair was 8–13 months, and repaired by Langenbeck’s method. All cases of two groups underwent PESQ (Perceptual Evaluation of Speech Quality) perception scoring by speech therapists before and after the operation. The speech assessors were blinded. The details including average and range of PESQ score were shown in Table 3. The clarity of pronunciation and nasalization in all cases improved compared to before the surgery. The oral shape and pronunciation of the patients also improved after the surgery. There was no significant difference at all aspects between the two groups. Throughout the 8-12month follow-up period, all wounds healed successfully without complications, such as infection, necrosis, perforation, or multiple fissures.

**Table 3:** Statistical analysis of speech quality for opposite rotation of BMMF + MPF and double-flap methods

	opposite rotation of BMMF and MPF	double-flap methods	Total	*p*-value
PESQMOS of Pre-operation				0.85
Average	1.3	1.4	2.7	
Range	0.5–1.8	0.5–1.5	0.5–1.8	
PESQMOS of post-operation				0.098
Average	3.5	3.4	6.9	
Range	1.8–3.5	1.9-3.0	1.8–3.5	

*PESQ *Perceptual Evaluation of Speech Quality

*P* > 0.05, No significant difference

### Typical case

**Fig. 3:**
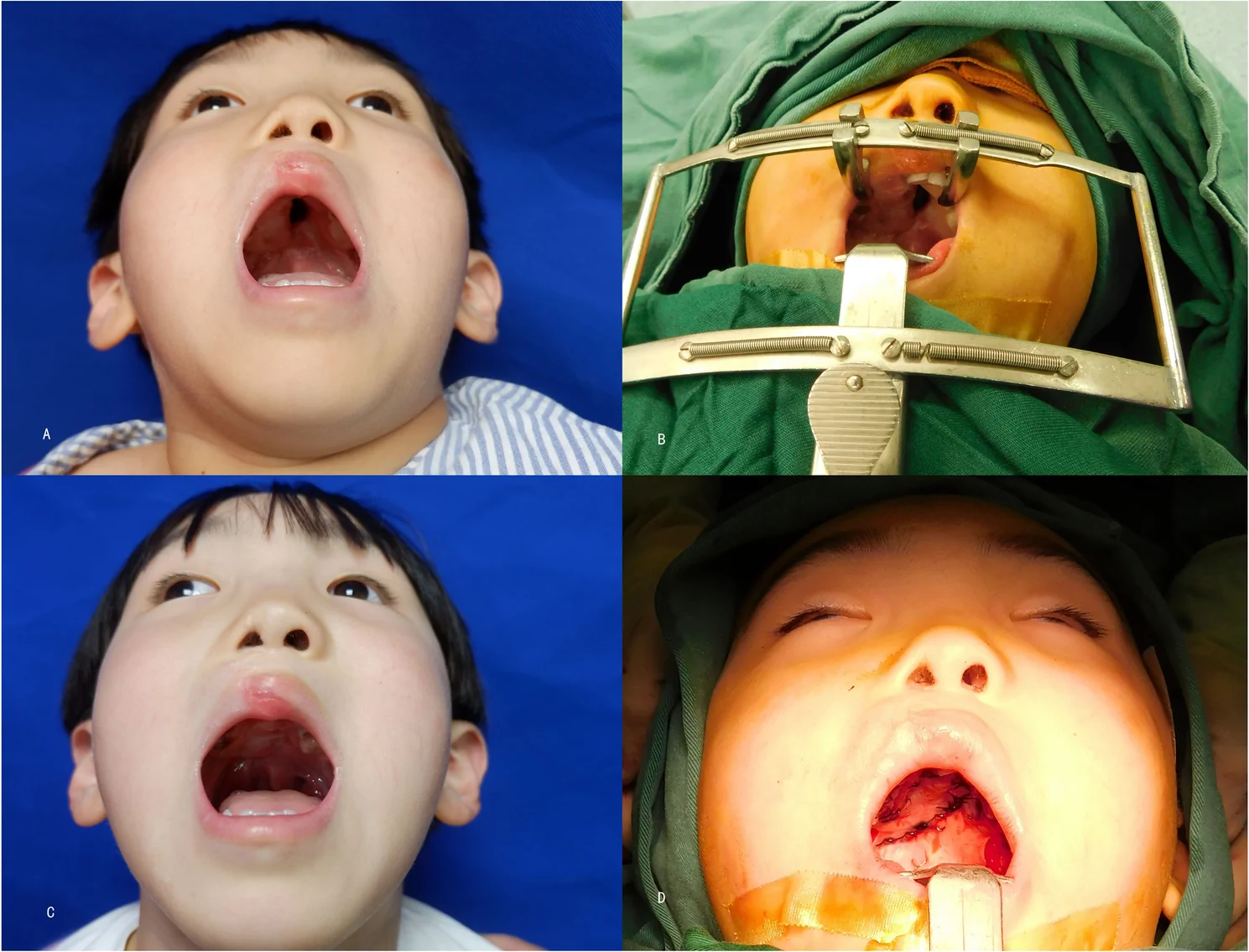
Typical case. **A** Preoperative Phase **B** Intraoperative Phase **C** 6-Month Follow-Up Phase **D** Completion of Intraoperative Repair

A 12-year-old male, who had undergone cleft lip repair at 3 months of age and palate repair at 1 year old, presented with an anterior palatal fistula resulting from an unsuccessful procedure. The patient sought medical examination at our hospital, displaying an overall satisfactory condition. A 25 mm x 10 mm fistula was observed on the right side of the middle palate, connecting to the nasal cavity. Preoperative examination confirmed the repair of the palatal fistula using the opposite rotation of the BMMF and MPF. The palatal fistula was successfully repaired by staggered rotation using the buccinator myo-mucosal flap and mucoperiosteal flap. The operation yielded success, and the patient exhibited a favourable postoperative recovery at the 6-month follow-up. Refer to Fig. 3A, B, C and D for illustration.

## Discussion

Congenital cleft lip and palate represent common malformations in children, and cleft palate significantly influences jaw bone development, language, and dietary aspects. Surgical intervention serves as the primary treatment for cleft palate; however, postoperative palatal fistula remains a prevalent complication [6], manifesting in different regions of the palate [7]. The recurrence rate of anterior palatal defect repair is high [8]. One of the reasons is that the blood supply of the used flap for repair is problematic, leading to recurrence. The blood supply to the palate is provided by the palatine major artery and the palatine minor arteries of the soft palate. The Mucoperiosteal flap we used directly receives blood supply from the palatine major artery. We chose the buccal mucosa flap as a local flap, but we also included a part of the buccal muscle, so the ratio of the length to width of the flap can reach 3:1 without causing necrosis.

Repairing an AFP poses challenges due to the presence of scarring on both sides of the palate behind the teeth and the limited availability of surrounding soft tissue for reconstruction. Insufficient tissue further complicates the repair process, presenting a dilemma for pediatric plastic surgeons. Currently, no standardised approach exists for palatal fistula repair, and the chosen method varies based on the location and size of the fistula. Various techniques, such as local tissue flaps [9, 10]and L Flap [11], prostheses utilizing original cleft palate incisions, tongue flaps [12], closure of palatal fistula with local double-breasted mucoperiosteal flaps [13], buccinator mucosal flaps [14], buccal fat pad flaps [15], and forearm radial free flaps [16], have been reported. Each method has its own set of advantages and disadvantages. Larger flaps with abundant blood supply are preferable for repairing palatal fistulas, but they may impede openings and impact eating and pronunciation in postoperative patients, particularly younger children who may exhibit reduced cooperation and an increased risk of avulsion, necessitating secondary pedicle repair. Free skin flaps, such as forearm flaps, necessitate an additional surgical site and leave a scar at the donor site, thereby complicating the procedure and resulting in different morphological function of the flap compared to mucosal tissue. Some reports have suggested the use of buccal fat pad flaps and buccinator mucosal flaps in adjacent flap areas for straightforward repairs, but these methods have limitations when addressing larger palatal fistulas. Other articles have mentioned the use of acellular dermal matrix [17] for palatal fistula repair, demonstrating promising results, though clinical applications have been less reported. Zielinska-Kazmierska [18] proposed repairing fistulas using a three-layered closure of oronasal fistulas. Abdaly [19] repaired palatal fistulas and palatopharyngeal insufficiency using buccinator flaps, with a recurrence rate of 4% for palatal fistulas. No cases of fistula recurrence, infection, flap ischemia or necrosis, or donor-site morbidity were observed during follow-up sessions. Rothermel [20] reported a toolbox of surgical techniques for palatal fistula repair and introduced the systematic repair of palatal fistulas. Reddy [21] suggested reducing recurrent palatal fistulas by using an antibiotic pack on the hard palate after fistula closure. Zheng [22] repair palatal fistula using free flap. These practices have shown effectiveness in preventing recurrent PF and repairing the PF is also a difficult task. The repair of the anterior palatal fistula is more challenging than that of other areas. Recent articles have reported the methods of Gingivoperiosteoplasty [23], Modified Buccal Myomucosal Flap [24] and Nasal Septal Flaps [25].All have achieved good results.

In our study, we investigated a technique for repairing APF, with a particular focus on utilizing adjacent palatal tissue that provides sufficient flap tissue for repair, particularly from the buccal region. Consequently, we devised a method involving the opposite rotation of the buccinator myo-mucosal flap and mucoperiosteal flap, coupled with the reversal lining of the hard palatal mucoperiosteum flap, to address large anterior palatal fistulas. This approach retains the use of local tissue flaps but represents an improvement over traditional local tissue flap repair method. The lining of the mucoperiosteal flap was facilitated using the local mucoperiosteal flap of the palatal margin, characterized by limited movement but effective suturability despite its brittle nature. Subsequently, the BMMF was rotated; however, direct suturing of the palate was impeded by a middle segment. To overcome this obstacle, we transferred the mucosa from the palate to the buccal mucosa, facilitating the closure of the fistula by suturing the tissues together without tension.

We posit that the method of opposite rotation of the BMMF and MPF, in conjunction with the reversal lining of the hard palatal mucoperiosteum flap, offers several advantages in palatal fistula repair: (1) It exhibits a high success rate for wide cracks in the front of the hard palate, including alveolar clefts on the palate’s side. (2) The pedicled BMMF provides ample tissue and is easily sutured. (3) The operative technique is straightforward and easily mastered. (4) PF at the junction of the hard and soft palate can also be effectively treated. (5) In the event of fistula recurrence, a substantial quantity of superior tissue is available, ensuring sufficient tissue for subsequent repairs.

### Study limitation

Due to the limited number of cases wherein this surgical method was employed, further validation with a larger sample size is imperative. Additionally, The study has the limitations due to the insufficient information for the VPI assessment, the clear description of innovative methods for the challenges of previously described article, a more comprehensive statistical analysis is essential to corroborate the efficacy of the method. The mucosa of the alveolar ridge is covered by the buccal mucosa, making it a weak foundation for future teeth roots and not a suitable alveolar ridge for keratinized mucosal area after bone grafting. Nevertheless, this technique remains an optimal approach for the treatment of anterior palate fistulas. Under appropriate selection criteria, the repair of palatal fistulas using the opposite of the BMMF and MPF, along with the rotation of the hard palatal mucoperiosteum flap flip lining, achieves a high success rate and is easily mastered, thereby meriting broader adoption. In the future, we will further refine the restrictive conditions section, incorporating factors such as small sample size, lack of random grouping, and short follow-up period. We will also study the potential impact of this method on future bone transplants.

## Conclusion

Opposite rotation of BMMF and MPF represents a novel method for repairing APF following cleft palate surgery, and low comorbidity. It can be used in large anterior palatal defect repairs and is applicable to all ages of anterior palatal fistula cases, and is a method of treated anterior palatal fistula.

## Data Availability

The datasets used or analyzed during the current study are available from the corresponding author on reasonable request.

## Abbreviations

BMMF: Buccinator myo-mucosal flap
MPF: Mucoperiosteal flap
APF: Anterior palatal fistula
PF: Palatal fistula
